# Enhanced remediation of U(vi) ions from water resources using advanced forms of morphologically modified glauconite (nano-sheets and nano-rods): experimental and theoretical investigations†

**DOI:** 10.1039/d4ra05514d

**Published:** 2024-09-03

**Authors:** Mostafa R. Abukhadra, Aya Fadl Allah, Mohamed Shaban, Noof A. Alenazi, Haifa A. Alqhtani, May Bin-Jumah, Ahmed A. Allam

**Affiliations:** a Materials Technologies and Their Applications Lab, Geology Department, Faculty of Science, Beni-Suef University Beni Suef City Egypt Abukhadra89@Science.bsu.edu.eg; b Geology Department, Faculty of Science, Beni-Suef University Egypt; c Department of Chemistry, Faculty of Science, Beni-Suef University 62514 Beni-Suef City Egypt; d Department of Physics, Faculty of Science, Islamic University of Madinah Madinah 42351 Saudi Arabia mssfadel@aucegypt.edu; e Department of Chemistry, College of Science and Humanities in Al-Kharj, Prince Sattam bin Abdulaziz University Al-kharj 11942 Saudi Arabia; f Department of Biology, College of Science, Princess Nourah bint Abdulrahman University P. O. BOX 84428 Riyadh 11671 Saudi Arabia; g Department of Biology, College of Science, Imam Mohammad Ibn Saud Islamic University Riyadh 11623 Saudi Arabia; h Department of Zoology, Faculty of Science, Beni-Suef University Beni-suef 65211 Egypt

## Abstract

Two forms of morphologically transformed glauconite (GL) involved exfoliated nanosheets (EXG) and nanorods (GRs), which were synthesized by facile exfoliating and scrolling modification under sonication. The two advanced forms (EXG and GRs) were applied as enhanced adsorbents for U(vi) ions and compared with using raw glauconite. The developed GRs structure displays higher saturation retention properties (319.5 mg g^−1^) in comparison with both EXG (264.8 mg g^−1^) and GL (237.9 mg g^−1^). This enhancement is assigned to the noticeable increment in the surface area (32.6 m^2^ g^−1^ (GL), 86.4 m^2^ g^−1^ (EXG), and 123.7 m^2^ g^−1^ (GRs)) in addition to the surface reactivity and exposure of effective siloxane groups. This was supported by the steric investigation based on the isotherm basics of the monolayer model of one energy site. The steric functions declared a strong increase in the density of the existing effective uptake receptors throughout the modification stages (GRs (112.1 mg g^−1^) > EXG (87.7 mg g^−1^) > 72.5 mg g^−1^ (GL)). Also, each active site can be filled with 4 U(vi) ions, donating the parallel orientation of these ions and the operation of multi-ionic mechanisms. The energetic functions, either the uptake energy (<13 kJ mol^−1^) or Gaussian energy (<5 kJ mol^−1^), validate the retention of U(vi) by physical reactions. These reactions displayed spontaneous properties and exothermic behaviors based on the investigated thermodynamic functions, including entropy, enthalpy, and internal energy. The structures also showed significant recyclability, indicating potential application on a realistic and commercial scale.

## Abstract 2

Two forms of morphologically transformed glauconite (GL) involved exfoliated nanosheets (EXG) and nanorods (GRs), which were synthesized by facile exfoliating and scrolling modification under sonication.

## Graphical abstract

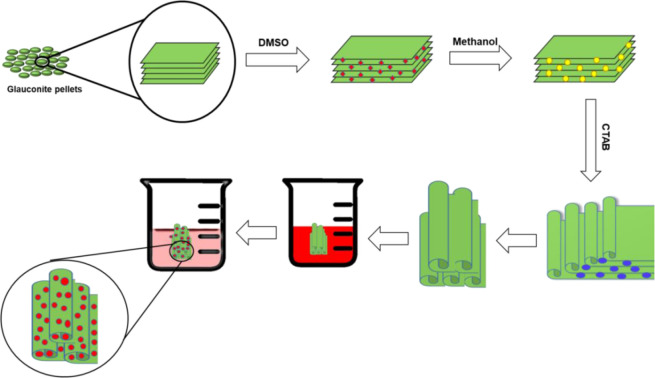

## 1 Introduction

The chemical contamination of water supplies, as well as the resulting detrimental effects on human well-being and the natural environment, pose serious challenges that pose a serious risk to humanity's future security.^1^ Unregulated and uncontrolled discharge of toxic sewage through mining, agricultural operations and other industries is an ongoing crisis.^2,3^ This is the root cause of the toxins present in the water today, as well as the subsequent environmental problems. The widespread distribution of toxic metals in lakes and rivers, either as soluble ions or complexes with other compounds, poses serious hazards to both the ecosystem's balance and human well-being.^2,4^ Researchers have classified all these pollutants as extremely hazardous, non-biodegradable, and cancer-triggering agents, which frequently accumulate inside the bodies of people and animals.^4–6^ Mining, mining-related industries, and nuclear fuel production are significant sources of many potential hazards and radioactive ions, such as uranium, radium, cesium, barium, strontium, and thorium.^7–9^

The nuclear power industry is closely associated with the formation of a significant quantity of radioactive byproducts and hazardous waste.^10–12^ Uranium is an essential component in the production of nuclear power and is widely recognized for its radiological properties and potential to contaminate water supplies.^13,14^ It has been established that uranium ions, especially the U(vi) type, have strong mobility and solubility. As a result, they are excessively seeping into water sources, posing a threat to both biodiversity and human well-being.^15,16^ The injection of U(vi) as a contaminant into human food supplies triggers liver damage, kidney failure, and ultimately death. Also, U(vi) contaminants greatly slow down the growth rates of organism embryos, which have negative effects on the ability of many species to survive and reproduce.^15^ The US Environmental Protection Agency set the upper limits of U(vi) in drinking water, surface water, and groundwater at 30 μg L^−1^, 50 μg L^−1^, and 2000 μg L^−1^, respectively.^9,17^

Various studies have suggested the use of adsorption by innovative materials as a cost-effective, efficient, secure, easily accessible, and recyclable approach for decontaminating various species of water pollutants including zeolite, clay based adsorbents, hydroxyapatite, TiO_2_, activated carbon, diatomite, and mesoporous silica.^16,18–20^ The selectivity of an adequate absorbing material is influenced by many aspects, including overall production costs, manufacturing techniques, precursor accessibility, adsorption effectiveness, recyclable potential, retention rate, biodegradability, uptake selectivity, security, durability, and reactivity.^20,21^ Consequently, a comprehensive assessment has been conducted for developing innovative adsorbents employing readily available and cost-effective components prevalent in natural resources.^1,22,23^ It is highly encouraged to utilize the established adsorbents derived from natural resources, including numerous varieties of minerals and rocks, as they provide great environmental and financial advantages.^16^ The geometrical aspects of synthetic materials have a significant impact on their biological, chemical, and physical qualities. The geometry of a material is vital in establishing its surface area, its adsorption effectiveness, and the availability of its binding sites.^24^ The application of nanomaterials with one-dimensional frameworks, including nanorods or nanotubes, alongside their two-dimensional morphological shapes as nano-sheets, has been recommended for various possible applications because of their excellent surface area, more effective dispersing qualities, and significant reactive interfaces.^25–27^

Subsequently, clay-based one-dimensional and two-dimensional nanoparticles were successfully produced as an enhanced adapted version of clay, possessing distinctive surface area alongside dispersion characteristics.^27,28^ This was mainly accomplished by the use of simple sonication-induced chemical-based exfoliation techniques to form single nano-sheets, complemented by scrolling steps to form the target nano-rods or tubes. Consequently, semicrystalline particulates were formed, consisting of single or multiple clay layers exhibiting distinctive exterior reactivity, dispersion effectiveness, surface area, oxidation properties, porosity, and adsorption characteristics.^27,29,30^ Nevertheless, the majority of investigations performed on the scraping and scrolling of clay minerals are mainly restricted to kaolinite or bentonite. No prior investigations were conducted to examine different types of clay minerals, especially glauconite. Glauconite is a prevalent mixed-layered clay mineral prevalent in nature, characterized by its potassium–ferric phyllosilicate framework, denoted as (K, Na) (Fe^3+^Fe^2+^, Al, Mg)_2_(Si,Al)_4_O_10_(OH)_2_.^31,32^ Glauconite is constructed from alternative layers of illite and smectite units, consisting of an alumina di-octahedral subunit sandwiched in between a pair of silica tetrahedron subunits. These successive layers additionally encompass interstitial K^+^ ions. Glauconite, as a mineral, possesses extensive resources, is affordable, and has a chemical composition that contains metals, has an appealing geometry, possesses an extensive surface area, demonstrates potential catalytic capacities, and has an elevated capacity for the exchange of ions.^33,34^ Hence, the mechanism of glauconite's transformation into nano-sheets and nanorods or tubes through exfoliating and scrolling will lead to the formation of a new framework that displays distinctive physicochemical characteristics along with excellent adsorption capacity whenever employed in decontaminating various water pollutants.^35–37^

In recent years, it has been established that synthetically produced clay nano-sheets, nanoscrolls, nanorods, and nanotubes are innovative and efficient adsorbents. These materials have a remarkably high surface area, a well-developed porous framework, and significant reactivity.^35^ Unfortunately, no studies have yet explored the adsorption efficacy of glauconite-based one-dimensional and two-dimensional structures for the successful removal of uranium ions as technique to follow the influence of glauconite obtained nanostructure morphology on its adsorption characteristics. The adsorption qualities were evaluated through comprehensive investigations, taking into account key variables and theoretical analysis. Theoretical analysis was conducted, examining both classic and advanced models to determine the saturation adsorption capacity, effective receptor density, quantity of immobilized ions per site, binding energy, and thermodynamic functions.

## 2 Experimental work

### 2.1. Materials

The glauconite mining deposits have been collected from the El-Gedida region in the El-Bahariya Oasis, located in the Western Desert of Egypt. The sample being investigated exhibited the following chemical composition based on the XRF analysis: SiO_2_ (52.2%), MgO (3.53%), Fe_2_O_3_ (23.14%), CaO (0.27%), K_2_O (6.48%), Al_2_O_3_ (6.12%), Na_2_O (0.08%), SO_3_ (0.17%), P_2_O_5_ (0.10%), MnO (0.01%), TiO_2_ (0.11%), and 7.8% loss on ignition (L. O. I.). Dimethyl sulfoxide (DMSO) (>99.5%), cetyltrimethylammonium bromide (CTAB) (>98%), NaOH pellets (97%), and methanol (>99.9%) have been purchased from Sigma-Aldrich and Egypt and were employed during the glauconite conversion procedures. The adsorption studies implemented standardized solution of uranium (U(vi)) (UO_2_(NO_3_)_2_ in HNO_3_ 2–3%) and had been purchased through the Sigma-Aldrich Company in Egypt.

### 2.2. Synthesis of glauconite exfoliated sheets (EXG) and nano-rods (GRs)

The production of the EXG has been performed using the documented steps by Abukhadra *et al.*^35^ Approximately 40 g of the initial glauconite as a raw mining mineral had been finely pulverized and then mixed with 200 mL of a diluted DMSO solution (80% DMSO with 10% water). The mixture was vigorously stirred for a period of 72 hours. This technique plays a key role throughout the breaking steps of the chemical bonds, especially the hydrogen bonds that exist between the successive layers of clay, particularly the illite subunits found in the mixed framework of glauconite with smectite. Subsequently, the resulting product underwent five cycles of washing using methanol, each of which lasted around 20 minutes. This process resulted in the formation of methoxy glauconite with a considerable exfoliation effect on its structural sheets (Mth/EXG) and organophilic characteristics. To successfully exfoliate the glauconite sheets, the resulting Mth/EXG portions underwent an immersion step within a water-based solution of CTAB to be an expanding agent (60 g within 200 mL of distilled water) at ambient temperature while being continuously stirred for 48 hours at 1000 rpm. Subsequently, the apparatus was subjected to ultrasound treatment over a duration of 96 hours using an ultrasound generator with a power supply of 240 W. This was done to ensure that the exfoliation of the glauconite silicate layers underwent an effective rolling process into rod-like structures. Following that, the end rod-like materials (GRs) had been extracted by filtering employing Whatman filter paper, repeatedly rinsed utilizing distilled water, and subsequently dried for 12 h at 60 °C.

### 2.3. Characterization instruments

The crystal structure and crystalline characteristics were evaluated and investigated by employing X-ray diffraction (XRD) patterns obtained *via* the PANalytical-Empyrean X-ray diffractometer. The determination limits of the 2 theta angles using the diffractometer extend from 0 to 70°. The alteration in the fundamental chemical groups throughout the fabrication stages was assessed employing a Shimadzu FTIR-8400S spectrometer, which encompasses the measurement range from 400 to 4000 cm^−1^. The surface features of the emerging materials, alongside the original glauconite, were examined by employing a Gemini Zeiss Ultra 55 scanning electron microscope. Prior to imaging, the exteriors of the materials under investigation were prepared by coating them with gold sheets by spraying. Furthermore, the interior frameworks and scrolled characteristics were further examined by means of HRTEM photographs, which were obtained utilizing a transmission electron microscope (JEOL-JEM2100) with an accelerated voltage of 200 kV. The porosity extents and specific surface area were measured using a surface area analyzer (Beckman Coulter SA3100) after evacuating any gases from the samples. The analysis has been completed, implementing the standard N_2_ adsorption and desorption isotherms.

### 2.4. Batch adsorption experiments

The adsorption experiments of U(vi) by raw glauconite (GL) as well as the synthesized EXG and GRs have been performed in batch mode, implementing the effects of pH (2–8), U(vi) content (50–400 mg L^−1^), and retention time (30–880 min). The experiments had been performed in three separate experiments, utilizing a constant volume of 200 mL and adsorbent dose of 0.2 g L^−1^. The adsorption equilibrium experiments were evaluated at various temperature settings, specifically 293 K, 303 K, and 313 K. Following the completion of each examination's equilibrium period, the treated solutions were filtered using Whatman filter paper (40 μm) to exclude any mixed particles of the adsorbents and determine the remaining contents of U(vi) ions. The residual levels of U(vi) were measured by applying inductively coupled plasma mass spectrometry (PerkinElmer). The results were subsequently employed to calculate the adsorption capacities of GL, EXG, and GRs, following eqn (1). The U(vi) standard that was utilized during the measurement procedures were purchased from Merck Company (Germany) and then certified by the National Standard and Technology Institute (NIST). The characters *Q*_e_, *C*_o_, *C*_e_, *V*, and *m* in the formula represent the adsorption capacity (measured in mg g^−1^), the starting levels (measured in mg L^−1^) of the metallic ions, the residual concentrations (measured in mg L^−1^) of the U(vi) ions, the volume (measured in mL) of the aqueous solutions contaminated with metals that were tested, and the dose (measured in mg) of GL, EXG, and GRs used.1
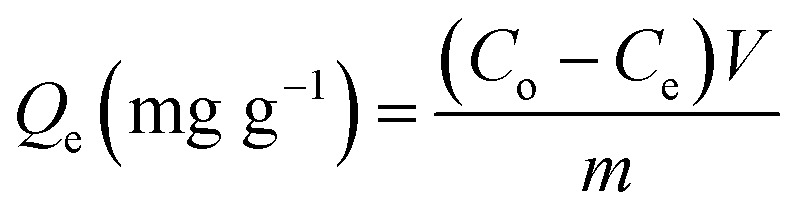


### 2.5. Conventional and modern equilibrium investigations

The adsorption of U(vi) using GL, EXG, and GRs has been described using well-established traditional kinetics, classic equilibrium, and updated isotherm investigations in accordance with the theoretical statistical physics hypothesis (Table S1†). The kinetic and conventional isotherm modeling have been assessed employing the non-linear fitting levels of the retention data of U(vi). The evaluation implemented the parameters of the coefficient (*R*^2^) (eqn (2)) and Chi-squared (*χ*^2^) (eqn (3)). The nonlinear fitting qualities with the modern isotherm models' descriptive equations and the remediation results of U(vi) have been examined using the determination coefficient (*R*^2^) and root mean square error (RMSE) (eqn (4)). The variables *m*′, *p*, Qi_cal_, and Qi_exp_ in the equation correspond to the outcomes of metal retention, parameters affecting metal retention, predicted capacities of metal retention, and determined capacities of metal retention, respectively.2
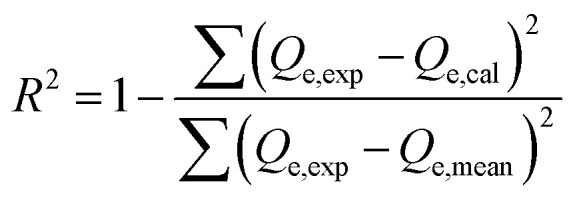
3
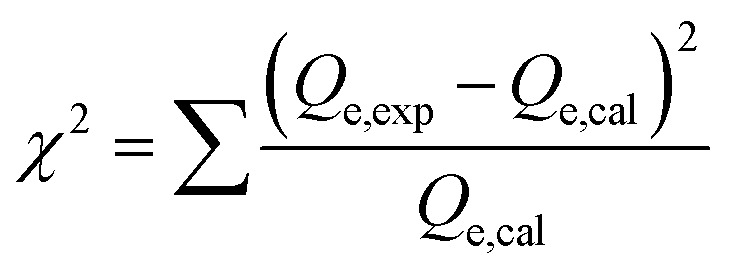
4
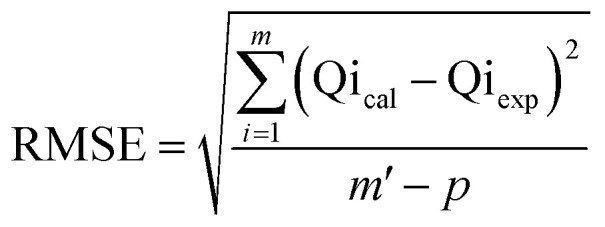


## 3 Results and discussion

### 3.1. Characterization of the used adsorbents

**Fig. 1:**
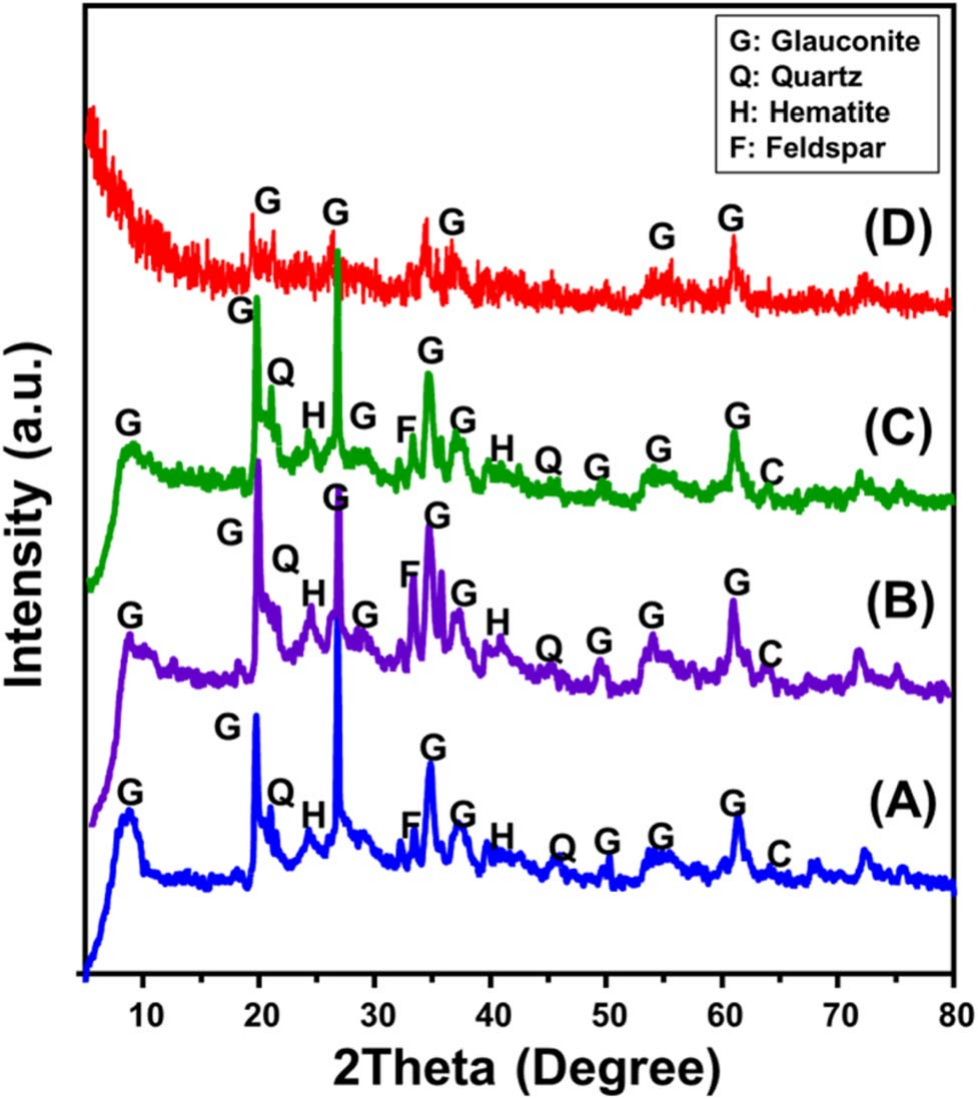
XRD patterns of glauconite (GL) (A), DMSO/G (B), methoxy exfoliated glauconite (EXG) (C), and synthesized GRs (D).

The XRD patterns of raw glauconite, produced GRs, and intermediate materials were analyzed to investigate their structural features (Fig. 1). The distinctive pattern of raw glauconite indicates that the main phase comprises a high K_2_O glauconite (1 M-glauconite poly-type) that has a structurally ISII-ordered form, along with specific impurities that include different minerals such as hematite and quartz (Fig. 1A).^38^ The peaks corresponding to glauconite were seen at angles of 8.67°, 19.72°, 26.70°, 34.78°, 37.17°, and 61.31° a with a basal spacing value equal to 10.18 Å (ICSD 166961) as shown in Fig. 1A (ref. 38–40). Following the addition of DMSO, the prominent peaks exhibited distinct variations to 8.01°, 19.54°, 26.5°, 34.5°, 36.8°, and 61.1° (Fig. 1B). The introduction of DMSO and its expansion impact on the *d*-spacing value (11.2 Å) seem to have caused significant deformation in the glauconite structural units. Similar results were obtained for Mth/EXG, with major peaks shifting to 7.47°, 19.52°, 26.50°, 34.40°, and 35.57° in addition to considerable elevation in the interlayer spacing value to 11.8 Å indicating a strong exfoliation influence (Fig. 1C). The pattern of synthetic GRs indicates a major deformation of the crystal and structural constituents of glauconite, resulting in its transition into a semi-crystalline or partly amorphized framework (Fig. 1D). The primary peaks exhibited a substantial reduction and were identified just as relics, validating the effective process of exfoliation and scrolling.

**Fig. 2:**
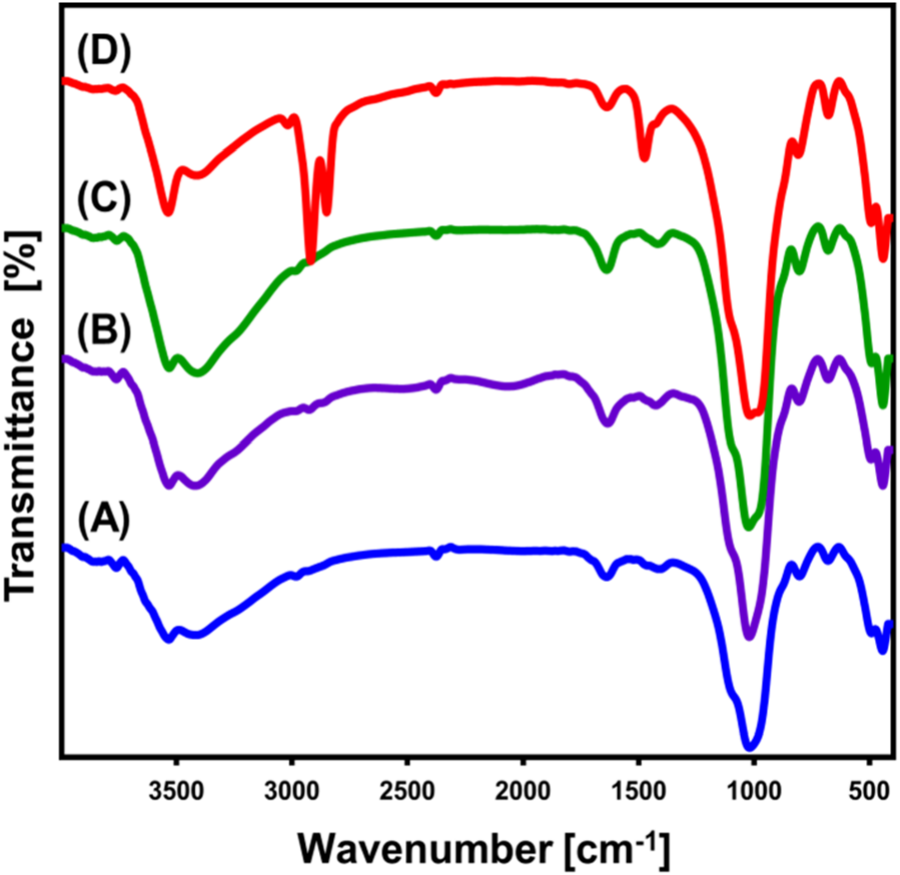
FT-IR spectra of glauconite (GL) (A), DMSO/G (B), methoxy exfoliated glauconite (EXG) (C), and synthesized GRs (D).

The FT-IR spectrum has been used to track and evaluate the chemistry and structural variations resulting from the modifying steps (Fig. 2). The spectrum analysis of untreated glauconite accurately reveals its definite composition as a clay mineral with an aluminosilicate structure. These include Si–O–Si (445.6 cm^−1^), Si–O–Fe^3+^ (493 cm^−1^), Si–O and/or OH (681 cm^−1^), Fe_2_^3+^OH/Fe^2+^Fe^3+^OH (804 cm^−1^), Si(Al)–O–Si (1020 cm^−1^), interlayer water (1639 cm^−1^), and OH of adsorbed water and/or skeletal metal hydroxides (3532 cm^−1^) (Fig. 2A).^41–43^ The existence of iron at an adequate level has been verified by the detected bands around 800 cm^−1^ and 490 cm^−1^, which aligns with the findings of the XRF chemical analysis (Fig. 2A). The DMSO/G spectrum (Fig. 2B), along with Mth/G (Fig. 2C) particulates, shows no significant changes compared to the untreated material, and there is no indication of any organic compounds being present. The main essential groups' identifying bands exhibit a little deviation from their exact positions, potentially indicating the structural influence of the integrated organic molecules of DMSO and methanol (Fig. 2B and C). In addition, there is a little split for the distinguishable band of Si(Al)–O–Si at about 1000 cm^−1^. This confirms the distortion of glauconite's structural constituents, particularly the alumina octahedron and silica tetrahedron units, attributed to the dislocation and separation of the silicate layers forming the exfoliated product (EXG).^44^ After the synthesis of GRs, the absorption spectra of the basic structural units of glauconite showed a considerable variation (Fig. 2D). Moreover, the splitting of the realized Si(Al)–O–Si band at about 1000 cm^−1^ verified a noticeable rise in distortion and exfoliating effectiveness (Fig. 2D). Additionally, other bands were also detected around 1475 cm^−1^ (methylene group), 2850 cm^−1^ (symmetrical CH_2_), and 2919 cm^−1^ (asymmetrical CH_2_), suggesting the presence of organic remnants correlated with the CTAB molecules that were employed in the transformation processes^35,45^ (Fig. 2D).

**Fig. 3:**
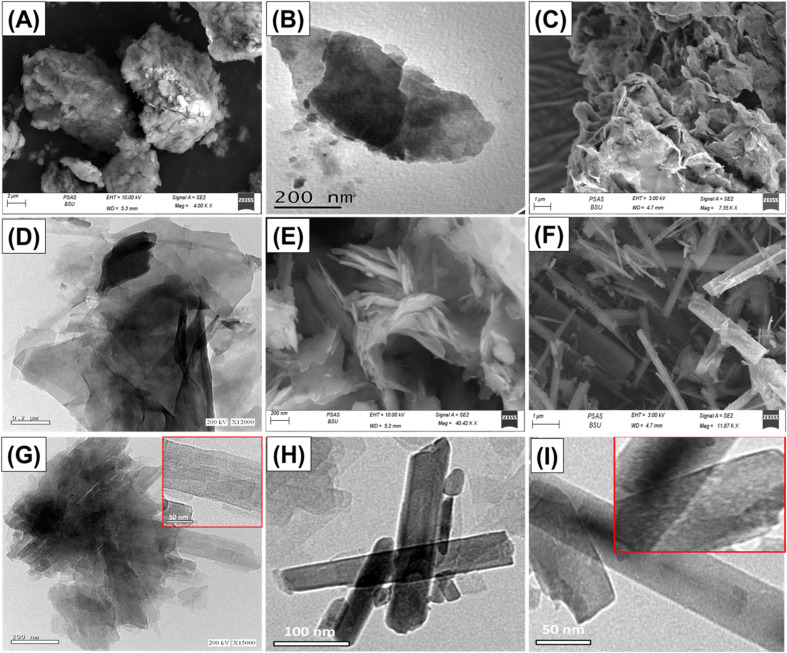
SEM image of glauconite (GL) (A), HRTEM image of glauconite (B), SEM image of exfoliated glauconite (EXG) (C), HRTEM image of EXG particles (D), SEM image of partially rolled GL (E), SEM image of well-formed GRs (F), HRTEM images of GRs with lattice figure structures (G), well developed scrolled single layered GRs with porous structure (H) and (I).

The synthetic products were also verified based on the SEM and HRTEM investigations. The untreated glauconite exhibits the characteristic compact and clustered form of glauconite, which is frequently encountered as packed and compacted layers (Fig. 3A and B). After embedding DMSO and methanol molecules between them, these layers successfully peeled off and separated from each other (Fig. 3C). After the incorporation of the extra alcohol molecules, the exfoliation behavior became much stronger, and the glauconite particles generated separate layers that overlapped and sometimes developed curvature platelets resembling cornflakes (Fig. 3C). The HRTEM images confirm the noticeable features. After the exfoliation, the particles transformed into separate sheets in the presence of the bended particles (Fig. 3D). After subjecting the glauconite sheets to the CTAB scrolling process, they exhibited considerable bending, which resulted in an arched shape resembling a curvature (Fig. 3E). The noticed particulates underwent a complete transformation into rod-like nanoparticles by extending the time of scrolling implemented through the ultrasonic supplier (Fig. 3F). The length of these particles varied between 150 nm and 5 μm, while their width ranged from approximately 25 nm to 200 nm. The HRTEM images confirmed the reality that the glauconite grains displayed a peeling-off tendency and started to roll, undergoing a transformation into multi-layered, rod-like nanostructures with noticeable lattice finger features (Fig. 3G). The extended duration of scrolling, especially when coupled with ultrasound, significantly compresses the resulting rod structure, which exhibits a smooth and cylindrical form, suggesting that each successive layer had been rolled independently into a single rod (Fig. 3H). Therefore, there is a lack of indications or proof substantiating the lattice finger features that have been earlier established. However, the high magnification images on the outer surface of the rods clearly revealed their highly porous properties, resulting in a framework with ribbon-like forms and properties (Fig. 3I). The marked changes in the surficial morphologies resulted in a strong impact on the surface area. The measured surface areas of GL, EXG, and GRs are 32.6 m^2^ g^−1^, 86.4 m^2^ g^−1^, and 123.7 m^2^ g^−1^, respectively.

### 3.2. Adsorption studies

#### 3.2.1. Effect of pH

**Fig. 4:**
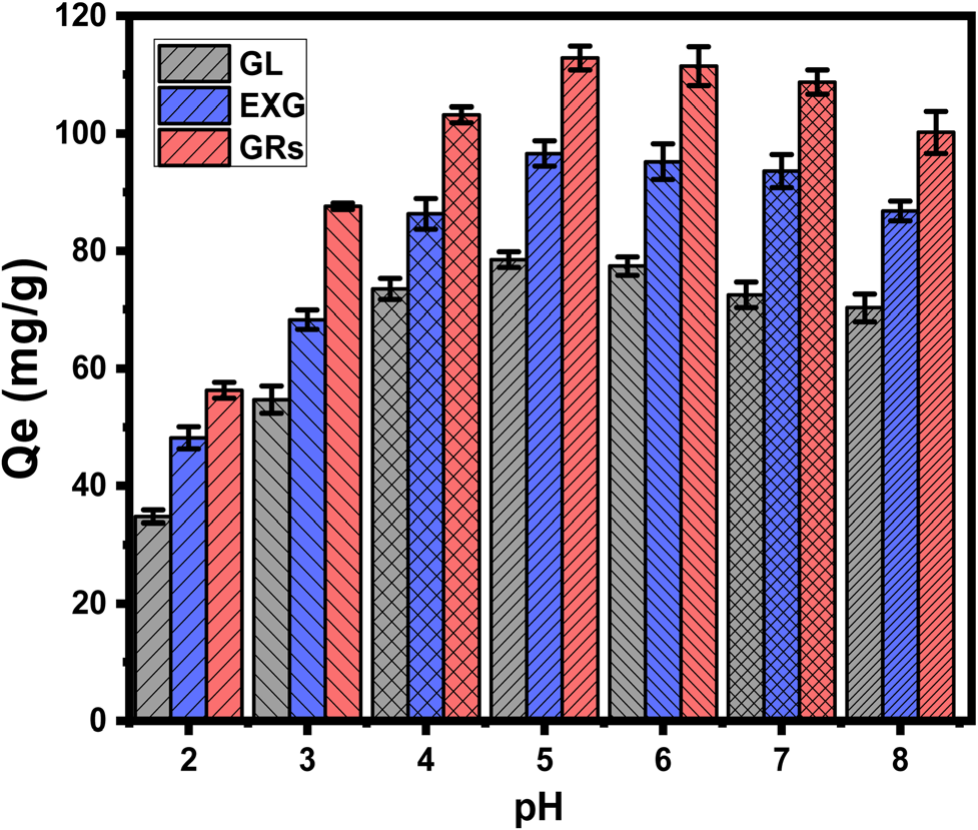
Effect of the solutions pH on the retention performances of U(vi) using GL, EXG, and GRs.

Adsorption of U(vi) was studied using GL, EXG, and GRs as effective adsorbents throughout a pH range of 2 to 8. The previously described experiments were performed after adjusting the values of the effective parameters, including the U(vi) content (100 mg L^−1^), the contact duration (120 minutes), the used adsorbent dose (0.2 g L^−1^), the treated volume (200 mL), and the operating temperature (20 °C). The binding effectiveness of U(vi) that was detected using GL, EXG, and GRs exhibits noticeable improvements whenever the pH of the contaminated solutions rises from pH 2 (34.8 mg g^−1^ (GL), 48.2 mg g^−1^ (EXG), and 56.3 mg g^−1^ (GRs)) to pH 5 (78.5 mg g^−1^ (GL), 96.6 mg g^−1^ (EXG), and 112.8 mg g^−1^ (GRs)) (Fig. 4). Following that, an apparent rise in pH caused a significant decrease in the established performance of U(vi) uptake by GL as well as EXG and GRs up to a pH of 8 (Fig. 4). Thus, the three structures possess the capacity to serve as efficient adsorption agents in real removal processes of U(vi) from wastewater, corresponding to the pH range of 6 to 9 specified by the US Environmental Protection Agency (EPA) for treating wastewater from industries.^46^ The observed behaviors indicate a strong correlation between the pH and the ionizing characteristics of U(vi) ions, together with the electrostatic charges on the surfaces of GL, EXG, and GRs.^9^

Based on the speciation behavior of uranium as soluble ions at different pH, at pH values close to or beneath 4.3, U(vi) predominantly occurs in a monomeric form as UO_2_^2+^, along with lower amounts of UO_2_(OH)^+^. Under pH levels higher than 5, U(vi) can be encountered in both colloidal and oligomeric forms, which include (UO_2_)_2_(OH)_2_^2+^, (UO_2_)_4_(OH)^7+^, and (UO_2_)_3_(OH)^5+^. The major forms under alkaline situations are the negatively charged forms, which include UO_2_(OH)^3−^ and (UO_2_)_3_(OH)^7−^.^9,16,47^ Thus, at lower pH settings, a considerable degree of competition and repulsion reactions involving H^+^ (or H_3_O^+^) and UO_2_^2+^ occurred at the interaction sites of GL, EXG, and GRs. This phenomenon demonstrated a gradual decrease in its influence until it reached a pH level of 5. Whenever the pH level rises above 5, the exteriors of GL, EXG, and GRs accumulate an elevated amount of negative electrical charges. This leads to significant repellent interactions with the negative U(vi) types that have been established at such pH settings.^12,48,49^ The GL, EXG, and GRs interfaces displayed negative electrical charges owing to the high concentration of hydroxyl groups on their interfaces. At acidic pH levels, the existence of hydronium ions (H^+^) induces positive charges on their surfaces, consequently impeding the removal of positively charged uranium(vi) ions. Therefore, the recognized experimental results and the previously mentioned speciation properties demonstrate that the exterior electronegativity of GL, EXG, and GRs significantly influences the retention of U(vi) in moderately acidic and marginally alkaline situations. These conditions intensify the impact of electrostatic attraction, resulting in a higher total retention rate compared to a strongly acidic setting. Consequently, it was established that GL, EXG, and GRs had a perfect pH level of 5 for U(vi) retention. The previous finding were supported with the determined values of pH value of zero point charge (pH_(pzc)_). The determined pH_(pzc)_ values during the uptake of U(vi) using GL, EXG, and GRs are pH 4.8, pH 5.3, and pH 4.2, respectively. The surficial charges beyond these values are mainly negative which induce the electrostatic attractions of the existed uranium species (UO_2_^2+^) up to pH 6. Also, during pH levels below these values, the surficial charges on the surface of the adsorbent are mainly positive which reduce the interaction of the existed uranium species.

#### 3.2.2. Effect of retention interval

**Fig. 5:**
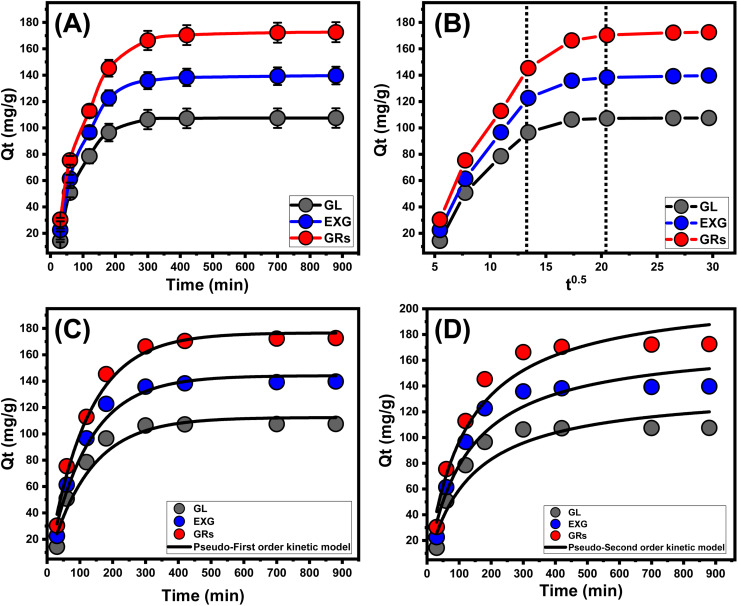
Experimental impact of contact duration on the retention performances of U(vi) (A), the plotted intra-particle diffusion curves for the retention of U(vi) using GL, EXG, and GRs (B), fitting of the determined retention behaviors with pseudo-first order model (C), and fitting of the determined retention behaviors with pseudo-second order model (D).

An examination was conducted to explore the retention characteristics of GL, EXG, and GRs in terms of duration. The time frame of the test varied between 30 and 880 minutes. After verifying the levels of essential parameters at specific values such as metal concentration (100 mg L^−1^), pH 5, temperature (293 K), volume (200 mL), and quantity (0.2 g L^−1^), the effectiveness of GL, EXG, and GRs in removing U(vi) can be verified by the significant increase in both the quantity of the adsorbed metal and the detectable rates of removal during the experiments (Fig. 5A). Additionally, it is crucial to recognize that the experiment's period has a significant influence on the detected changes in the retention properties, which could extend as long as 300 minutes (Fig. 5A). Nevertheless, there has been no discernible variation or improvement noticed in the rate at which the metal ions are removed or the amounts of ions retained after the predetermined duration (300 min). Previous findings indicate that GL, EXG, and GRs have the ability to serve as adsorption agents for U(vi) and reach an equilibrium state within 180 minutes. The retention capacities of U(vi) using GL, EXG, and GRs at equilibrium were 107.5 mg g^−1^, 139.7 mg g^−1^, and 172.6 mg g^−1^, respectively (Fig. 5A). During the early phases of the investigation, there were notable improvements and increased rates of removal for U(vi) by GL, EXG, and GRs, as well as higher amounts of retained ions. The improvements were ascribed to the prevalent presence of interacting and free receptors throughout the framework of GRs.^16^ As the length of the examination increased, there was a noticeable reduction in the quantity of accessible sites. The main factor responsible for this behavior may be attributed to the prolonged retention of U(vi) at the existing and free binding sites, resulting in a reduction in the overall number of unoccupied sites. Consequently, there has been a substantial drop in the rate at which U(vi) ions were adsorbed after certain duration. Furthermore, the use of GL, EXG, and GRs demonstrated little enhancement or consistent characteristics in the adsorption of U(vi), indicating a states of stability or equilibrium situations. The equilibrium phases of GL, EXG, and GRs can potentially be established by completely occupying all the active receptors, consequently inhibiting further adsorption of U(vi) to their interface.^2^

#### 3.2.3. Kinetic studies

##### 3.2.3.1. Intra-particle diffusion behavior

Analyzing the characteristics of intra-particle diffusion curves has the ability to reveal the progressive mechanisms and retention tendencies of U(vi) utilizing GL, EXG, and GRs. The provided curves show three distinct sections with different gradients (Fig. 5B). The assessed curves exhibit displacements from their initial positions, indicating the simultaneous occurrence of various adsorption reactions in addition to the diffusion modes of U(vi).^9,50^ The uptake activities typically involve three main phases: (1) the reactions between the ions of the metals and the available receptors across the outer surfaces of GL, EXG, and GRs (boundary); (2) the layered uptake of the ions (internal adsorption) along with the diffusive effects of these ions; and (3) the effects of the stable state and saturating situations.^51^ The preliminary findings of the analysis reveal that the main mechanisms responsible for anchoring U(vi) onto the exterior interfaces of GL, EXG, and GRs (external retention) were the most significant pathways encountered during the various stages of retention activities (Fig. 5B). The effectiveness of retention of U(vi) during this stage depends on the total number of sites that exist throughout the interfaces of GL, EXG, and GRs.^19^ By increasing the duration whenever all exterior sites had been completely occupied (Fig. 5B); the effectiveness of further layered adsorption techniques was immediately established.^51^ In addition, the effects of U(vi) diffusion pathways are considered throughout this stage. Whenever an equilibrium state is achieved, the last U(vi) retaining mechanisms by GL, EXG, and GRs exert an extensive effect. This indicates that the U(vi) ions, which were efficiently retained, occupied all of the available binding sites.^9,18^ During this step, the elimination of U(vi) is promoted by molecular and interionic attraction processes.^48^

##### 3.2.3.2. Kinetic modeling

Kinetic modeling of the retention of U(vi) is crucial for investigating time-dependent consequences and interpreting the physical mechanisms associated with them, including mass transfer or chemical mechanisms that regulate adsorption effectiveness.^52^ The standard kinetic concepts of pseudo-first-order (P. F.) (Fig. 5C and Table 1) and pseudo-second-order (P. S.) (Fig. 5D and Table 1) numerical models have been employed to analyze the kinetics of the removal activities of U(vi). The P. F. modeling had been employed to analyze the kinetics of the retention behaviors throughout equilibrium conditions to illustrate the correlation between the rate by which the ions completely fill the interaction sites of binding and their overall quantities. The P.S. concepts could potentially be employed to demonstrate the correlation between the properties of evaluated adsorbents during a specific period of time. The correlation degrees between the retaining characteristics of the water-soluble ions and kinetic concepts were examined using nonlinear fitting parameters that corresponded to the appropriate equations. This evaluation has been conducted in relation to the two assumptions. By examining the correlation coefficients (*R*^2^) and Chi-squared (*χ*^2^) values, the most suitable levels of agreement have been determined (Table 1; Fig. 5C and D).

**Table 1:** The theoretical parameters of the assessed kinetic models

Model	Parameters	GL	EXG	GRs
Pseudo-first-order	*K*_1_ (1/min)	0.0080	0.0083	0.0082
	*Q*_e (cal)_ (mg g^−1^)	112.4	144.1	176.6
	*R* ^2^	0.95	0.97	0.98
	*χ* ^2^	1.77	0.98	0.63
Pseudo-second-order	*k*_2_ (mg g^−1^ min^−1^)	5.47 × 10^−5^	4.62 × 10^−5^	3.87 × 10^−5^
	*Q*_e (cal)_ (mg g^−1^)	137.9	174.9	213.4
	*R* ^2^	0.92	0.94	0.96
	*χ* ^2^	3.05	2.25	1.79

The fundamental hypotheses of the P. F. theory (Fig. 5C) provide better information on the binding reactions and retention activities of U(vi) applying GL, EXG, and GRs than the evaluated P.S. hypothesis (Fig. 5D). The *R*^2^ in conjunction with the *χ*^2^ data demonstrates the underlying kinetic characteristics (Table 1). The numerical modeling employing the P.F. model revealed that the hypothetical values of U(vi) adsorbed using GL, EXG, and GRs were 112.4 mg g^−1^, 144 mg g^−1^, and 176.6 mg g^−1^, respectively (Table 1). These results were in accordance with the quantities detected by experimentation. The reported alignment validates the previously established findings, which emphasize the better suitability of the P.F. hypothesis to represent the kinetic properties of the retention reactions of these ions (Table 1). According to the P.F. hypothesis, the key reasons for retaining U(vi) ions using GL, EXG, and GRs encompass physical processes such as van der Waals forces or electrostatic attraction.^53,54^ The studied kinetic modeling parameters also show an excellent degree of agreement with the P.S. concepts; however, a greater degree of agreement is achieved for the P.F. model. Previous studies have shown that particular chemical mechanisms, including complexation, ion exchange, and hydrophobic interactions, have the potential to enhance or have little influence on the uptake of U(vi) using GL, EXG, and GRs.^53^ Successive retention of physically adsorbed layers may be generated over the formerly formed chemically bound layers of U(vi).^55^

#### 3.2.4. Effect of U(vi) concentration

**Fig. 6:**
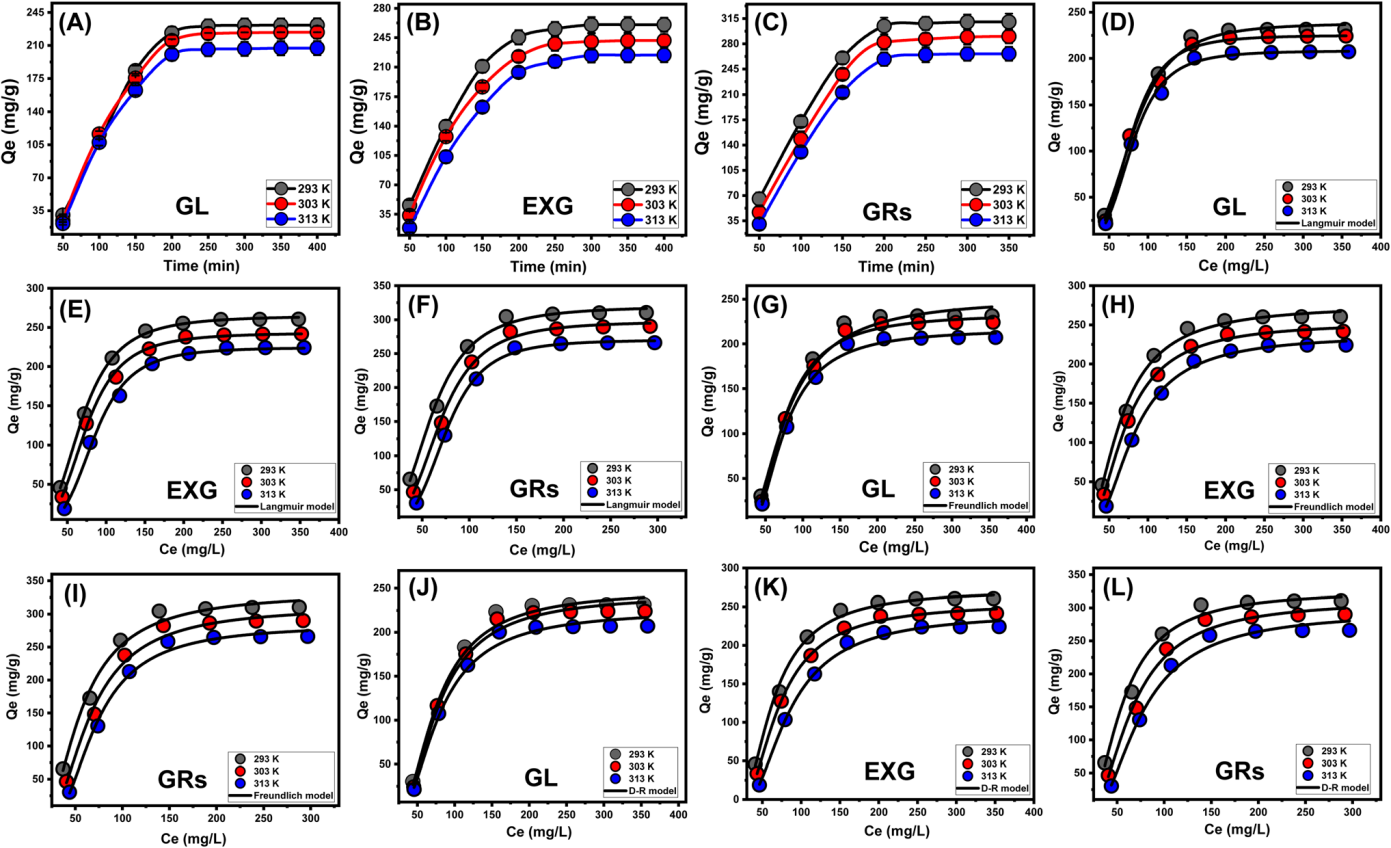
Experimental impact of starting contents of U(vi) on its retention performances ((A) (GL), (B) (EXG), and (C) (GRs)), fitting of the retention results with classic Langmuir model ((D) (GL), (E) (EXG), and (F) (GRs)), fitting of the retention results with classic Freundlich model ((G) (GL), (H) (EXG), and (I) (GRs)), and fitting of the retention results with classic D–R model ((J) (GL), (K) (EXG), and (L) (GRs)).

The experiment investigated the impact of the initial metal level on the removal efficiency of U(vi) employing GL, EXG, and GRs. It also studied the corresponding equilibrium conditions within the tested concentration range of 50 to 400 mg L^−1^. The parameters that affected the elimination of U(vi) were kept fixed at certain values. These values encompassed a total volume of 200 mL, a time frame of 24 hours, a pH of 5, a dose of 0.2 g L^−1^, and temperatures ranging from 293 K to 313 K. A relationship has been established between elevated concentrations of four types of metal ions and the reported rise in the quantities of U(vi), which are adsorbed using GL, EXG, and GRs (Fig. 6A–C). The rise in the levels of metal ions inside a certain volume significantly enhanced the diffusion and mobility properties of the soluble metals. This enhanced the ability to interact with a greater quantity of reacting retention sites, which are abundantly present across the exteriors of GL, EXG, and GRs. As a result, the effectiveness of U(vi) retention tendencies using GL, EXG, and GRs was significantly improved with the regular elevation of their starting contents.^18^ Nevertheless, this correlation is only observable under certain constraints for the concentrations of U(vi). After that, it seems that raising the starting levels of the metal does not have an impact on their binding efficiency and demonstrates their equilibrium uptake stages. Determining the equilibrium phases assists in estimating the highest possible retention efficiency of U(vi). The retention capacities of U(vi) using GL at temperatures of 293 K, 303 K, and 313 K were 231.5 mg g^−1^, 224.1 mg g^−1^, and 207.2 mg g^−1^, respectively (Fig. 6A). The observed adsorption possibilities using EXG were 260.5 mg g^−1^ at 293 K, 241.6 mg g^−1^ at 303 K, and 224.2 mg g^−1^ at 313 K (Fig. 6B). The measured values for GRs were 310.4 mg g^−1^ at 293 K, 290.2 mg g^−1^ at 303 K, and 266.1 mg g^−1^ at 313 K (Fig. 6C). The decrease in the elimination of the four ions using GL, EXG, and GRs at different temperatures suggests that the uptake reactions are exothermic. The significant enhancement in surface area and reactivity of the separated silicate layers may account for the enhanced effectiveness of EXG and GRs in removing U(vi) ions compared to GL particles. These layers possess semi-crystalline features and a significant number of chemically reacting sites, especially the siloxane groups.

#### 3.2.5. Classic isotherm models

Traditional equilibrium analyses were conducted to evaluate the distribution of water-soluble pollutants in aqueous solutions and the materials that adsorb them in the equilibrium state. Conventional equilibrium models have a significant impact on the elucidation of the mechanisms of the uptake processes. The frequently used isotherm functions provide useful insights into three important features including (a) the sorbate's affinities towards the adsorbent's reactive interfaces; (b) the hypothetical quantities of soluble chemical ions that might react with these surfaces; and (c) the maximal potential for binding to them. The study investigated the binding behavior of U(vi) ions by analyzing their equilibrium functions using the Langmuir (Fig. 6D to F), Freundlich (Fig. 6G to I), and Dubinin–Radushkevich (D–R) (Fig. 6J to L) isotherm models. The consistency between the assumed equilibrium hypotheses outlined in the aforementioned models and the measurable retention characteristics of U(vi) ions were evaluated by non-linear fitting techniques. The inquiry entailed analyzing the correlation coefficient (*R*^2^) and the Chi-squared (*χ*^2^) results, as shown in Table 2. The evaluation of *R*^2^ and *χ*^2^ indicates that the GL particles as well as EXG and GRs particles have a stronger tendency to adsorb the U(vi) ions with more agreement with Langmuir's theories rather than the Freundlich concept. This demonstrates that U(vi) ions display consistent and uniform tendencies for adsorption through the homogenously existing reactive and unbound sites of GL, EXG, and GRs particles. This leads to the formation of a solitary layer or monolayers comprised of adsorbed U(vi) ions.^53,54^ Furthermore, the examination revealed that particulates of GL, EXG, and GRs had favorable retention characteristics for U(vi) ions, as evidenced by the RL values that are less than 1 (Table 2).^18,19^ The theoretical study determined that the greatest adsorption capabilities (*Q*_max_) of U(vi) ions using GL were 238.4 mg g^−1^ at 293 K, 229.1 mg g^−1^ at 303 K, and 211.3 mg g^−1^ at 313 K. The computed values for EXG are as follows: 268.9 mg g^−1^ at 293 K, 245.1 mg g^−1^ at 303 K, and 226.5 mg g^−1^ at 313 K (Table 2). The predicted values for GRs are as follows: 320 mg g^−1^ at 293 K, 297.5 mg g^−1^ at 303 K, and 270.3 mg g^−1^ at 313 K (Table 2).

**Table 2:** The theoretical parameters of the assessed classic isotherm models

Material	Model	Parameter	Values		
			293 K	303 K	313 K
GL	Langmuir	*Q*_max_ (mg g^−1^)	238.4	229.1	211.3
		*R* ^2^	0.99	0.99	0.99
		*χ* ^2^	0.246	0.185	0.172
		*b* (L mg^−1^)	5.40 × 10^−7^	5.36 × 10^−8^	4.22 × 10^−8^
	Freundlich	1/*n*	0.47	0.39	0.39
		*k*_F_ (mg g^−1^)	251.1	233.4	216.2
		*R* ^2^	0.98	0.98	0.97
		*χ* ^2^	1.22	1.16	1.18
	D–R model	*β* (mol^2^ kJ^−2^)	0.0439	0.0459	0.0481
		*Q*_m_ (mg g^−1^)	248.14	242.9	225.2
		*R* ^2^	0.99	0.99	0.99
		*χ* ^2^	0.77	0.35	0.36
		*E* (kJ mol^−1^)	3.37	3.3	3.22
EXG	Langmuir	*Q*_max_ (mg g^−1^)	268.9	245.14	226.5
		*R* ^2^	0.99	0.99	0.99
		*χ* ^2^	0.016	0.125	0.237
		*b* (L mg^−1^)	2.78 × 10^−6^	6.45 × 10^−7^	5.99 × 10^−8^
	Freundlich	1/*n*	0.47	0.44	0.42
		*k*_F_ (mg g^−1^)	275	253	236.8
		*R* ^2^	0.988	0.98	0.98
		*χ* ^2^	1.22	1.07	1.03
	D–R model	*β* (mol^2^ kJ^−2^)	0.0315	0.0388	0.0436
		*Q*_m_ (mg g^−1^)	272.3	254.9	242.2
		*R* ^2^	0.99	0.99	0.99
		*χ* ^2^	0.22	0.07	0.13
		*E* (kJ mol^−1^)	3.98	3.56	3.38
GRs	Langmuir	*Q*_max_ (mg g^−1^)	320.1	297.5	270.3
		*R* ^2^	0.99	0.99	0.99
		*χ* ^2^	0.238	0.198	0.065
		*b* (L mg^−1^)	8.33 × 10^−6^	9.9 × 10^−7^	6.03 × 10^−8^
	Freundlich	1/*n*	0.48	0.45	0.40
		*k*_F_ (mg g^−1^)	332.5	311.4	283.3
		*R* ^2^	0.97	0.98	0.98
		*χ* ^2^	1.65	1.07	1.46
	D–R model	*β* (mol^2^ kJ^−2^)	0.0235	0.033	0.0438
		*Q*_m_ (mg g^−1^)	325.6	311.3	294.3
		*R* ^2^	0.99	0.99	0.99
		*χ* ^2^	0.62	0.61	0.56
		*E* (kJ mol^−1^)	4.61	3.89	3.38

The equilibrium parameters of the D–R model provide a comprehensive understanding of the energy variations exhibited by GRs particulates throughout the removal of U(vi) ions, independent of the particle's degree of homogeneity.^56^ The analysis of the D–R modeling results provides valuable knowledge about the estimation of Gaussian energies (*E*) and its function in recognizing the key mechanisms, regardless of whether they are chemical-based or physical-based activities. Energy levels corresponding to the retention activities can be categorized into three distinctive groups: that <8 kJ mol^−1^, those within 8 and 16 kJ mol^−1^, and those higher than 16 kJ mol^−1^. At these energy levels, the main mechanisms consist mostly of intense physical, weak chemical, or a combination of physical and chemical interactions, and significant chemical processes.^16^ The reported *E* values for U(vi) retention processes using GL, EXG, and GRs were found to be within the approved energy range for physical mechanisms (Table 2).

#### 3.2.6. Advanced isotherm models

**Fig. 7:**
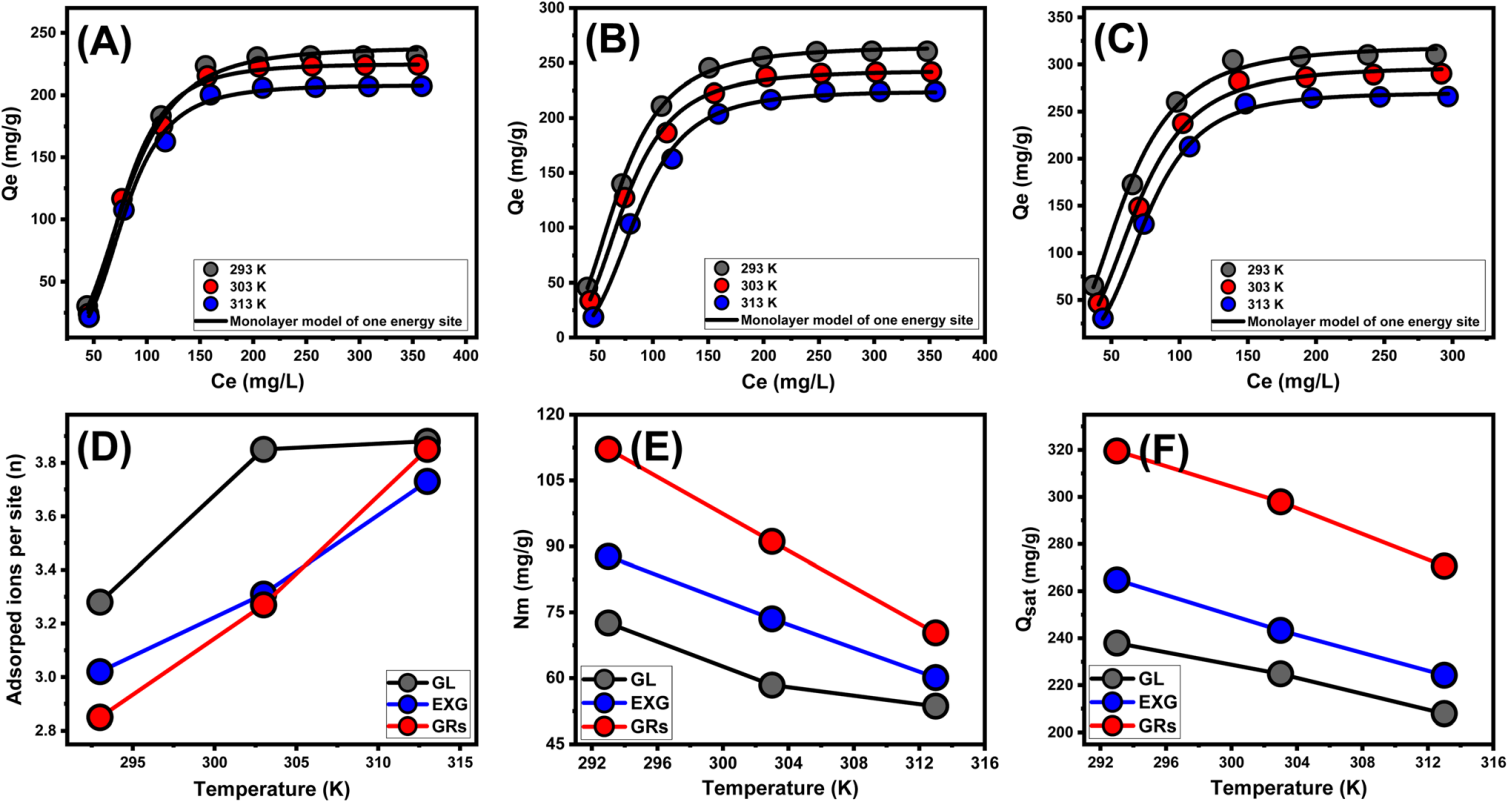
Fitting of the experimental results with advanced monolayer model of one energy site ((A) (GL), (B) (EXG), and (C) (GRs)), changes in the number of adsorbed U(vi) ions per site (D), changes in the occupied active sites density during the uptake of U(vi) (E), and changes in the saturation uptake capacities of U(vi) using GL, EXG, and GRs (F).

Using the statistical physics concepts for modeling the equilibrium properties of adsorption behaviors could offer a detailed examination of the particular characteristics of these reactions. The numerical models employed in the present analysis monitor the reactions between water-soluble pollutants and external reactive chemical groups. These chemical groups act as binding receptors on the exteriors of the material used for adsorption. The numerical formulas implemented in this investigation offer precise estimated parameters that properly describe the essential activities, involving energetic and steric aspects. The numerical simulations include several steric aspects, including the *N*_m_, which represents the overall quantity of occupied adsorption sites across the interaction frameworks of GL, EXG, and GRs. Moreover, the computations determine the number of metal ions (*n*) that a single receptor can adsorb, as well as the maximum retention capacity of U(vi) ions using GL, EXG, and GRs when they reach a fully saturated state (*Q*_sat_). The energetic factors include internal energy (*E*_int_), entropy (*S*_a_), retention energies (*E*), and free enthalpy (*G*). The previously mentioned hypotheses of the established models were evaluated using non-linear regression analyses. Multivariable nonlinear regression methods, in conjunction with the Levenberg–Marquardt iterative approach, effectively completed the prior inquiry. The fitting degrees accomplished were then used to evaluate and describe the adsorption remarks of these metal ions using GL, EXG, and GRs. The assignment was completed using the most analogous model, namely the monolayer model with a single active site (Fig. 7A–C). Table 3 shows the calculated parameters based on the evaluated model.

**Table 3:** The obtained steric and energetic parameters derived from advanced equilibrium modeling

		*n*	*N*_m_ (mg g^−1^)	*Q*_sat_ (mg g^−1^)	*C*_1/2_ (mg L^−1^)	Δ*E* (kJ mol^−1^)
GL	293 K	3.28	72.54	237.9	80.7	−11.24
	303 K	3.85	58.4	224.8	83.4	−11.71
	313 K	3.88	53.6	207.9	89.9	−12.29
EXG	293 K	3.02	87.7	264.8	68.9	−10.85
	303 K	3.31	73.5	243.3	74.35	−11.41
	313 K	3.73	60.1	224.2	85.65	−12.16
GRs	293 K	2.85	112.1	319.5	60.2	−10.52
	303 K	3.27	91.1	297.9	68.9	−11.22
	313 K	3.85	70.3	270.6	75.3	−11.82

##### 3.2.6.1. Steric properties

###### 3.2.6.1.1. Number of adsorbed metal ions per site (*n*)

The scientific findings from the *n* function provide significant insights into the arrangement of adhered U(vi) ions across the exterior surfaces of the GL, EXG, and GRs. This includes both the vertical and horizontal arrangements. Moreover, these findings have significant implications for understanding the processes that regulate binding reactions, such as multiple dockings or multiple interactions. The presence of many retention sites and the development of multi-anchorage or multi-docking processes significantly affect the sequestration of U(vi) ions. Retention activities with values below 1 suggest that these adsorbed ions are oriented horizontally. On the other hand, activities with quantities greater than 1 indicate the existence of U(vi) ions in non-parallel arrangements with vertical orientation. Removal strategies for such systems predominantly depend on multi-ionic pathways, in which a single site may accommodate numerous metal ions.^18,57^ The assumed quantities of *n*, representing the total number of metal ions occupied on a single retention site across the exterior of adsorbents, range from 3.28 to 3.88 for GL, from 3.02 to 3.73 for EXG, and from 2.85 to 3.85 for GRs (Fig. 7D and Table 3). The values above 1 indicate that all four varieties of metal ions have been retained by multi-ionic interactions. Every retention site within the GL, EXG, and GRs particulates demonstrated the ability to adsorb four ions of U(vi). These ions were organized in vertical arrangements with non-parallel characteristics. In terms of temperature effects, as the temperature rises from 293 K to 303 K, the estimated *n* levels of GL, EXG, and GRs for U(vi) ions significantly increase (Fig. 7D and Table 3). The hypothesized increase in the aggregating characteristics of U(vi) ions following their retention on the surface of GL, EXG, and GRs at elevated temperatures could potentially explain the observed findings.^58^

###### 3.2.6.1.2. Density of the active sites (*N*_m_)

The binding sites that were filled with U(vi) ions across the interfaces of GL, EXG, and GRs particles can potentially be precisely established by assessing the density of the active sites. The values that were determined for the computed *N*_m_ for GL at various temperature settings were 72.5 mg g^−1^ at a temperature of 293 K, 58.4 mg g^−1^ at a temperature of 303 K, and 53.6 mg g^−1^ at a temperature of 313 K (Fig. 7E and Table 3). The measured quantities of EXG were 87.7 mg g^−1^ at 293 K, 73.5 mg g^−1^ at 303 K, and 60.1 mg g^−1^ at 313 K. The assumed levels for GRs were 112.1 mg g^−1^ at 293 K, 91 mg g^−1^ at 303 K, and 70.3 mg g^−1^ at 313 K (Fig. 7E and Table 3). The findings confirm a significant increase in the total number of reactive sites following exfoliation modifications, which in turn leads to the development of separate nano-sheets and the subsequent wrapping of these layers into nano-rods with enhanced dispersion properties. The main reasons for these behaviors include a rise in surface area, enhanced exposure of existing functional active siloxane groups, and increased reactivity of the silicate interface as a consequence of transforming into semi-crystalline or amorphous materials. These modifications enhance the development of an interacting interface between soluble U(vi) ions and the exterior layers of EXG and GRs.

Regarding temperature impacts, the values of *N*_m_ on the surfaces of GL, EXG, and GRs during the retention reactions of these ions display reversible variations with the operating temperature (Fig. 7E and Table 3). The recognized profiles correspond to the earlier described behavior of *n*, as the enhanced aggregation properties of U(vi) lead to a reduction in the total quantity of filled sites. It appears that temperature influences the levels of activation of the already present retention sites, which could contribute further to illustrating this behavior.^1,58^ The investigation highlights how increasing temperatures have a negative effect on the number of occupied sites, either by eventually deactivating certain operating sites or by shortening the time needed for these sites to effectively adsorb these ions. Prior studies displayed similar tendencies, which could be ascribed to the hypothesized diffusion or desorption of metal ions that were previously bound onto their interfaces. The desorption behavior occurred as a consequence of the reduction in saturation limitations of warmed solutions.

###### 3.2.6.1.3. Adsorption capacity at the saturation state of (*Q*_sat_)

The saturated adsorption properties of GL, EXG, and GRs (*Q*_sat_) contribute to optimal retention of U(vi), as well as the highest level of tolerance. Two crucial factors affect the calculation of *Q*_sat_ values: the designated density of occupied sites (*N*_m_) and the total quantity of metal ions hosted within each individual site (*n*). The retention effectiveness of U(vi) by GL particles are 237.9 mg g^−1^ at a temperature of 293 K, 224.8 mg g^−1^ at 303 K, and 207.9 mg g^−1^ at 313 K. The maximum adsorption capacities using EXG were determined to be 247 mg g^−1^ at 293 K, 224 mg g^−1^ at 303 K, and 209.3 mg g^−1^ at 313 K (Fig. 7F and Table 3). The documented measurements for GRs are 319.5 mg g^−1^ at 293 K, 297.9 mg g^−1^ at 303 K, and 270.6 mg g^−1^ at 313 K (Fig. 7F and Table 3). The temperature-related negative effects indicate that the U(vi) retention processes using GL, EXG, and GRs are exothermic. These results suggest that elevated retention temperatures increase the percentage of thermal collisions, which reduces the binding efficiency of U(vi) ions.^57^ Furthermore, *Q*_sat_'s temperature-dependent observable properties reveal similarities to the activity described by *N*_m_ rather than *n*. The findings suggest that the extent of interacting receptors, rather than the specific binding capability of each individual receptor, primarily influences the efficiency of U(vi) retention by GL, EXG, and GRs.

##### 3.2.6.2. Energetic properties

###### 3.2.6.2.1. Adsorption energy

The observed changes in energy (Δ*E*) during the adsorption procedures of U(vi) ions could offer valuable insights into the mechanisms that underlie them, regardless of whether they correspond to physical or chemical processes. Physical activities have energies below 40 kJ mol^−1^, while chemical interactions have energies higher than 80 kJ mol^−1^. The binding energies serve as a useful metric for classifying different physically realized mechanistic processes. The specified binding mechanisms include hydrogen binding (that includes an energy below 30 kJ mol^−1^), dipole binding reactions (that include an energy range of 2–29 kJ mol^−1^), van der Waals forces (that include an energy range of 4–10 kJ mol^−1^), and hydrophobic binding (that includes an energy of 5 kJ mol^−1^). Eqn (5) had been employed to calculate the energies of the removal processes (Δ*E*) mathematically for U(vi) ions. This equation incorporates the solubility of the metal being investigated (*S*), the gas constant (*R* = 0.008314 kJ mol^−1^ K^−1^), the levels of metal ions under half-saturating conditions of GL, EXG, and GRs, and the temperature (*T*).^59^5
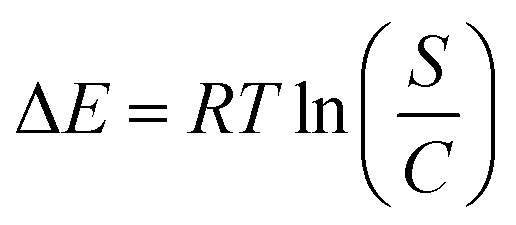


The energy values correlated with the removal of U(vi) ions range from −11.24 to −12.29 kJ mol^−1^ for GL, from −10.85 to −11.16 kJ mol^−1^ for EXG, and from −10.52 to −11.8 kJ mol^−1^ for GRs (Table 3). As a result, the key mechanisms that regulate the uptake of U(vi) ions using GL, EXG, and GRs are predominantly physical, involving dipole bonding (2–29 kJ mol^−1^) and hydrogen bonding (less than 30 kJ mol^−1^) in addition to the impact of electrostatic attractions and slight effect of van der Waals forces (4–10 kJ mol^−1^). Furthermore, the documented negative signals of the presented Δ*E* values while retaining U(vi) ions using GL, EXG, and GRs are in accordance with prior experimental findings that suggest the exothermic characteristics of these processes.

###### 3.2.6.2.2. Thermodynamic functions

***Entropy***: the entropy (*S*_a_) of U(vi) ions adsorption processes employing GL, EXG, and GRs reveals the distinct order and disorder features that characterize their exterior interfaces under different metal ion levels and temperatures. The properties of *S*_a_ were demonstrated by applying the results of eqn (6), which included the earlier determined values for *N*_m_ and *n* along with the expected levels of U(vi) ions during the half-saturation states of GL, EXG, and GRs (*C*_1/2_).6
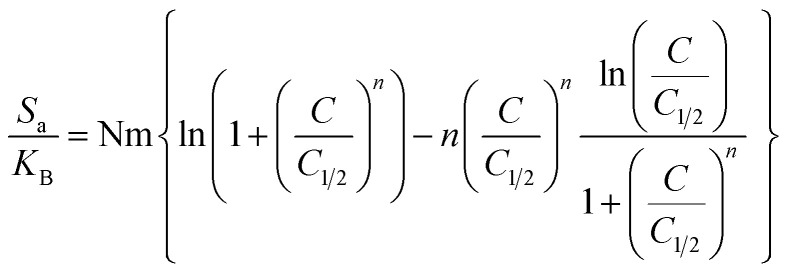


**Fig. 8:**
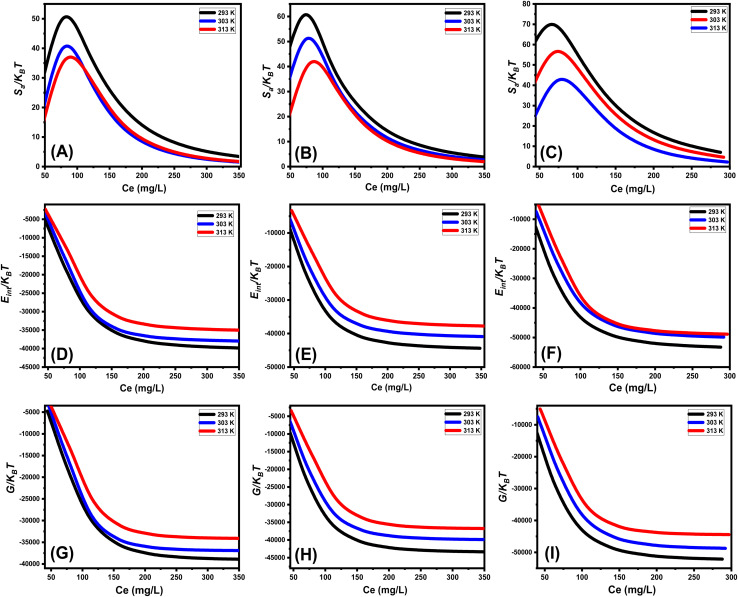
Changes in the behavior and values of thermodynamic functions during the retention of different concentrations of U(vi) at different temperature; entropy ((A)–(C)), internal energy ((D)–(F)), and free enthalpy ((G)–(I)).

Analysis of the obtained graphs demonstrates that as U(vi) ions were binding on GL, EXG, and GRs, specifically at high starting levels of these ions, there was a notable decrease in the entropy degree (*S*_a_) (Fig. 8A to C). These findings reveal a clear decrease in the disorder features that distinguish the contact surfaces of GL, EXG, and GRs as the contents of the U(vi) ions increase. This reflects the remarkable improvement in the successful adherence and bonding of U(vi) ions to the vacant and active receptors situated on the interface of GL, EXG, and GRs, despite their low contents.^59,60^ Following the uptake of U(vi) by GL, the greatest entropy degree was identified at equilibrium levels of 74.5 mg L^−1^ (293 K), 76.7 mg L^−1^ (303 K), and 78.5 mg L^−1^ (313 K) (Fig. 8A). The equilibrium levels of U(vi) ions corresponding to the maximum entropy using EXG were 73.9 mg L^−1^ at 293 K, 74.5 mg L^−1^ at 303 K, and 79.3 mg L^−1^ at 313 K (Fig. 8B). The adsorption processes using GRs exhibit the maximum entropy at concentrations of 65.5 mg L^−1^ (at 293 K), 70.3 mg L^−1^ (at 303 K), and 48.2 mg L^−1^ (at 313 K) (Fig. 8C). These equilibrium readings are largely approximated by the concentrations reported following the half-saturation phases of GL, EXG, and GRs. Therefore, the existence of remaining binding sites hinders the ability to bond extra metal ions during the docking process. Furthermore, the significant reductions recognized in the measured entropy levels indicate a substantial drop in the number of available sites, as well as a considerable fall in the mobility and diffusion properties of U(vi) ions.^61^

***Internal energy and free enthalpy***: The analysis of the internal energy (*E*_int_) as well as the free enthalpy corresponding to the binding behaviors of U(vi) ions using GL, EXG, and GRs was conducted in terms of the variations in the contents of the metals and operating temperature. The assessment was performed using eqn (7) and (8) using the predetermined values for *N*_m_, *n*, and *C*_1/2_, as well as the translation partition (*Z*_v_).^62^7
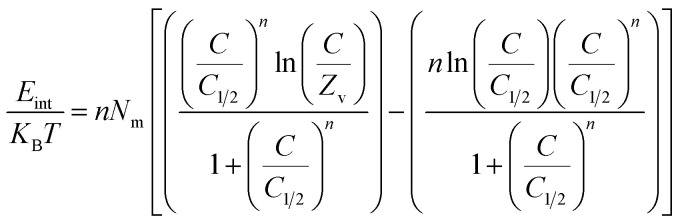
8
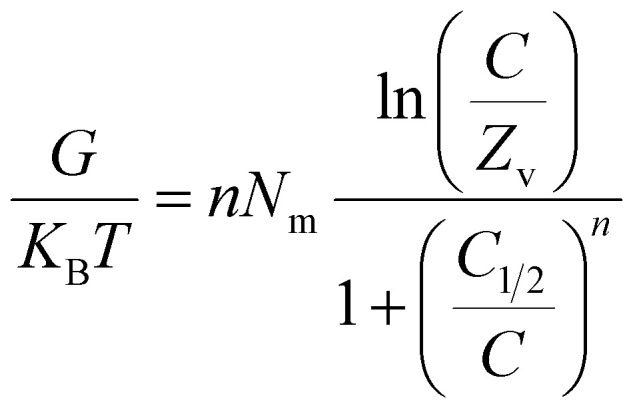


The analysis of fluctuations in *E*_int_ in relation to the removal activities of U(vi) ions, using GL, EXG, and GRs, shows negatively signed values. The results indicate a significant decrease in *E*_int_ as the temperature increases from 293 K to 313 K (Fig. 8D to F). This investigation confirms the spontaneous and exothermic nature of the U(vi) retention reactions using GL, EXG, and GRs. The enthalpy assessments and responses have the same characteristics and specifications as the internal energy. The G data exhibit adverse trends and demonstrate a reversible relationship with the particular binding temperature (Fig. 8G to I). This signifies a decrease in the feasibility aspects and affirms the exothermic nature and spontaneous characteristics of the retention reactions of U(vi) ions utilizing GL, EXG, and GRs.

#### 3.2.7. Recyclability

**Fig. 9:**
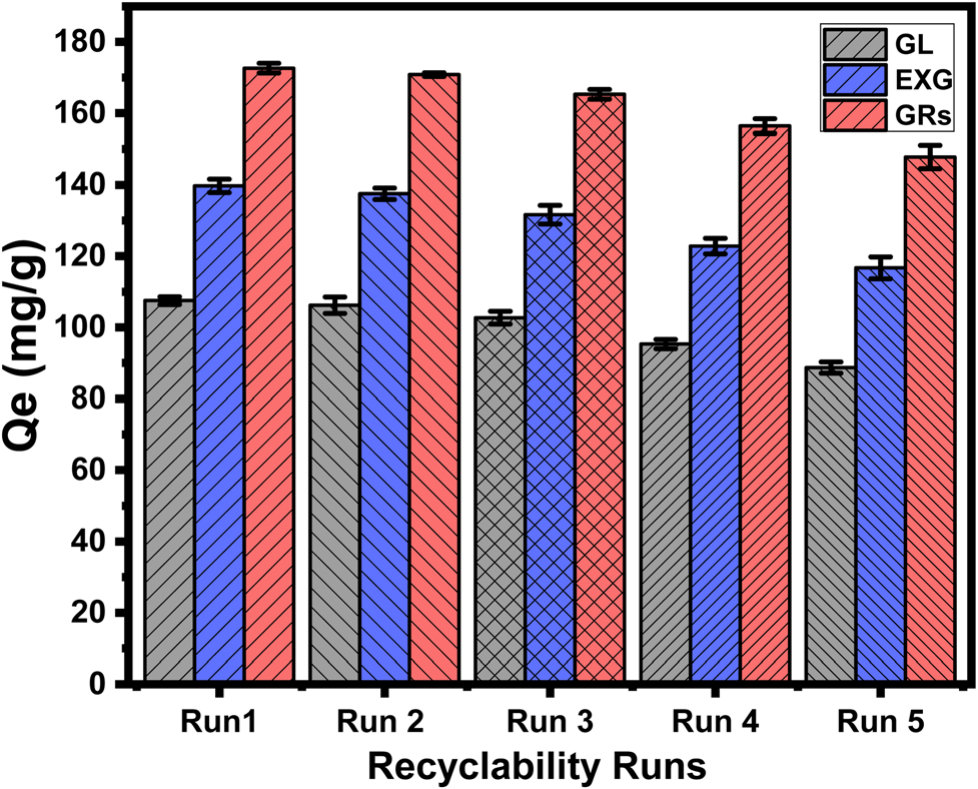
The recyclability properties of GL, EXG, and GRs during the adsorption of U(vi) ions.

The recyclability of GL, EXG, and GRs as adsorbents has a substantial impact on assessing the structure for commercial and actual applications. The regeneration methods implemented to clean the utilized GL, EXG, and GRs particles included a meticulous washing step using a 0.1 M NaOH solution (20 mL) at an ambient temperature of 50 °C over a period of 30 minutes using an orbital shaker. Following this, the collected particles were neutralized using distilled water and then dried for 12 hours at 50 °C. The U(vi) ions have undergone reusable adsorption examinations in controlled testing settings. The specified parameters were as follows: a total volume of 200 mL, an interval of 24 hours, a pH of 5, a metal content of 100 mg L^−1^, a dose of 0.2 g L^−1^, and a temperature of 293 K. The results highlight the excellent recyclability of GL, EXG, and GRs and their ability to be utilized repeatedly as adsorbents for U(vi) ions at the concentration level being tested (Fig. 9). The U(vi) removal efficiency realized using GL was 107.5 mg g^−1^ (Run 1), 106.2 mg g^−1^ (Run 2), 102.7 mg g^−1^ (Run 3), 95.3 mg g^−1^ (Run 4), and 88.7 mg g^−1^ (Run 5) (Fig. 9). The measured values for EXG in Run 1, Run 2, Run 3, Run 4, and Run 5 are 139.7 mg g^−1^, 137.5 mg g^−1^, 131.5 mg g^−1^, 122.8 mg g^−1^, and 116.7 mg g^−1^, respectively (Fig. 9). The documented results for GRs are 172.6 mg g^−1^ (Run 1), 170.8 mg g^−1^ (Run 2), 165.3 mg g^−1^ (Run 3), 156.4 mg g^−1^ (Run 4), and 147.8 mg g^−1^ (Run 5) (Fig. 9). The effectiveness of GL, EXG, and GRs as adsorbents for U(vi) ions decreased with the rise in the number of reuse and recycling rounds. This trend illustrates the ongoing formation of chemical complexes involving the essential effective chemical groups of GL, EXG, and GRs and the adsorbed U(vi) ions. These complexes negatively affect the overall quantity of available, free, and active sites after each recycling test.

#### 3.2.8. Comparison study

The retention values for U(vi) employing GL, EXG, and GRs were compared with the other adsorbents described in the literature. Table 4 shows that the GRs structure is more efficient than the unprocessed glauconite (GL) and the exfoliated derivative (EXGL), as well as most of the other adsorbents listed in the table involving certain metallic oxides, whether in composites or pure forms. This highlights the significance of using synthesized GRs and EXGL as cost-effective, secure, and highly efficient adsorbents that are suitable for practical U(vi) ion treatment activities.

**Table 4:** Comparison between the retention capacities of the studied structures and other materials in literature

Adsorbent	*Q*_(max)_ (mg g^−1^)	Reference
Amidoxime chitosan/bentonite	49.09	63
Activated carbon	158	64
Fe_3_O_4_	125	65
Carbonaceous nanofibers	125	66
SBA-15	208	67
nZVI/CNF	54.95	68
Polyacrylamide/chelating sorbents	65.3	69
Montmorillonite-Fe_3_O_4_–TiO_2_	109.11	70
MnFe_2_O_4_	119.9	71
Nickel ferrite/graphene oxide	123	72
GO-MnO_2_	85.2	73
MCM-48	125	74
Graphene oxide nanosheets	97.5	75
Fe_3_O_4_@C	120.2	76
Fe_3_O_4_@TiO_2_	118.8	77
GL	**237.9**	**This study**
EXGL	**264.8**	**This study**
GRs	**319.5**	**This study**

## 4 Conclusion

The glauconite (GL) was successfully exfoliated into separated nano-sheets (EXG) and scrolled into well-developed nano-rods (GRs). The modified structures were characterized as enhanced adsorbents for U(vi) (319.5 mg g^−1^ (GRs), 264.8 mg g^−1^ (EXG), and 237.9 mg g^−1^ (GL)). The GRs product, followed by EXG, showed better results than the raw sample. This was illustrated based on the experimental characterization and theoretical findings of advanced isotherm modeling. The morphologically modified forms exhibited enhanced surface area (32.6 m^2^ g^−1^ (GL), 86.4 m^2^ g^−1^ (EXG), and 123.7 m^2^ g^−1^ (GRs)) and higher active site density (GRs (112.1 mg g^−1^) > EXG (87.7 mg g^−1^) > 72.5 mg g^−1^ (GL)) in addition to the surface reactivity and dispersion properties. The steric (number of adsorbed ions per site (*n* = 4)) along with energetic functions (uptake energy (<13 kJ mol^−1^)) suggested the retention of U(vi) by multi-ionic physical mechanisms. These mechanisms also occurred spontaneously with exothermic behavior, considering the thermodynamic findings.

## Data availability

The data will be available up on request to corresponding author.

## Conflicts of interest

There are no conflicts to declare.

## Supplementary Material

RA-014-D4RA05514D-s001
