# Supplementary material for: Enhanced remediation of U(vi) ions from water resources using advanced forms of morphologically modified glauconite (nano-sheets and nano-rods): experimental and theoretical investigations
Source: RSC Adv. 2024 Sep 3;14(38):28017–34. doi: 10.1039/d4ra05514d (PMC11369765; doi:10.1039/d4ra05514d)
Supplement: RA-014-D4RA05514D-s001 [file RA-014-D4RA05514D-s001.pdf]

Supplementary Information (SI) for RSC Advances.
This journal is © The Royal Society of Chemistry 2024

Table.S1. Nonlinear equations of kinetic, classic isotherm, and advanced isotherm models

Model	Equation	Parameters
**Kinetic models**		
**Model**	**Equation**	**Parameters**
**Pseudo-first-order**	$Q_t = Q_e\,(1 - e^{-k_1.t})$	Q_t (mg/g) is the adsorbed ions at time (t), and K_1 is the rate constant of the first-order adsorption (1/min)
**Pseudo-second-order**	$Q_t = \frac{Q_e^2 k_2 t}{1 + Q_e k_2 t}$	Qe is the quantity of adsorbed ions after equilibration (mg/g), and K_2 is the model rate constant (g/mg min).
**Classic Isotherm models**		
**Model**	**Equation**	**Parameters**
**Langmuir**	$Q_e = \frac{Q_{max}\, bC_e}{(1 + bC_e)}$	C_e is the rest ions concentrations (mg/L), Q_{max} is the theoritical maximum adsorption capacity (mg/g), and *b* is the Langmuir constant (L/mg)
**Freundlich**	$Q_e = K_f C_e^{1/n}$	K_F (mg/g) is the constant of Freundlich model related to the adsorption capacity and n is the constant of Freundlich model related to the adsorption intensities
**Dubinin–Radushkevich**	$Q_e = Q_m e^{-\beta\varepsilon^2}$	β (mol²/KJ²) is the D-R constant, ε (KJ²/mol²) is the polanyil potential, and Q_m is the adsorption capacity (mg/g)
**Advanced isotherm models**		
**Model**	**Equation**	**Parameters**
**Monolayer model with one energy site (Model 1)**	$Q = nN_o = \frac{nN_M}{1 + (\frac{C1/2}{C})^n} = \frac{Q_o}{1 + (\frac{C1/2}{C})^n}$	Q is the adsorbed quantities in mg/g<br>n is the number of adsorbed ion per site<br>Nm is the density of the effective receptor sites (mg/g)
**Monolayer model with two energy sites (Model 2)**	$Q = \frac{n_1 N_{1M}}{1 + (\frac{C_1}{C})^{n_1}} + \frac{n_2 N_{2M}}{1 + (\frac{C_2}{C})^{n_2}}$	Q_o is the adsorption capacity at the saturation state in mg/g<br>C1/2 is the concentration of the ions at half saturation stage in mg/L
**Double layer model with one energy site (Model 3)**	$Q = Q_o \frac{(\frac{C}{C1/2})^n + 2(\frac{C}{C1/2})^{2n}}{1 + (\frac{C}{C1/2})^n + (\frac{C}{C1/2})^{2n}}$	C1 and C2 are the concentrations of the ions at the half saturation stage for the first active sites and the second active sites, respectively<br>n1 and n2 are the adsorbed ions per site for the first active sites and the second active sites, respectively
**Double layer model with two energy sites (Model 3)**	$Q = Q_o \frac{(\frac{C}{C1})^n + 2(\frac{C}{C2})^{2n}}{1 + (\frac{C}{C1})^n + (\frac{C}{C2})^{2n}}$	

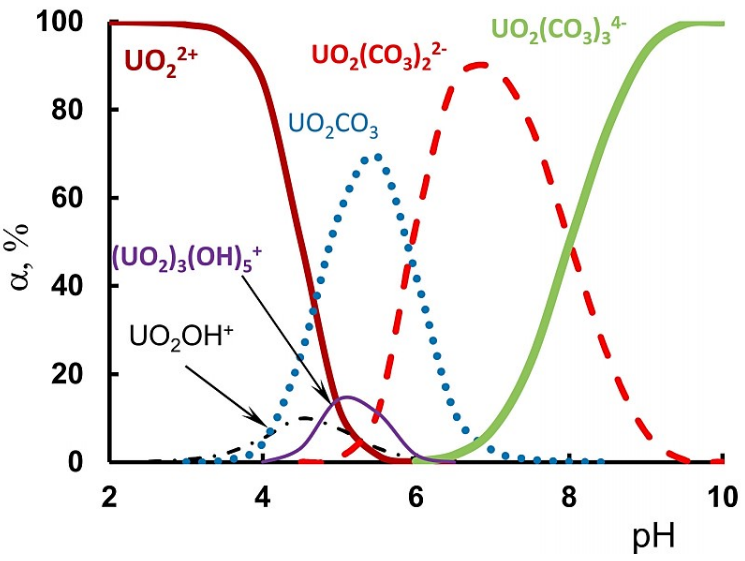

Fig.S1. The speciation diagram of uranium at different pH values [47]
